# Constructing and Implementing a Low-Cost On-Demand Morris Water Maze Platform

**DOI:** 10.21769/BioProtoc.5428

**Published:** 2025-09-05

**Authors:** Bradley Stinnette, Jeffrey M. Long, Craig Myrum

**Affiliations:** 1 Department of Biology, Loyola University Maryland, Baltimore, MD, USA; 2 National Institutes of Health (NIH), Laboratory of Behavioral Neuroscience, Neurocognitive Aging Section, National Institute on Aging, Baltimore, MD, USA

**Keywords:** Morris water maze (MWM), Memory, Spatial learning, Rats, Low-cost, Do-it-yourself (DIY), Cognitive aging, F344 rats

## Abstract

The Morris water maze (MWM) is one of the most widely used procedures to assess hippocampus-dependent spatial learning and memory in rodents. By varying test protocols, researchers can test several different domains of learning and memory. Over multiple testing days, animals learn to swim to a platform hidden just under the water surface by using the spatial relationship between distal cues and the platform. Probe trials, where the platform is rendered unavailable, measure rodents’ spatial bias for the area where the platform was previously located. The ability of researchers to control the availability of the platform “on-demand” offers both practical and methodological advantages. Despite MWM’s prominence in the field of behavioral neuroscience, the high cost of purchasing a commercial MWM package is often prohibitively expensive for many research labs, especially on-demand platforms. Here, we describe a low-cost strategy for a build-your-own MWM that includes a remote-controlled on-demand platform (~530 USD) and tank (~550 USD). It is our hope that disseminating low-cost strategies aimed at expanding access to high-quality research tools at underfunded research institutions will accelerate biomedical discovery and foster further innovation.

Key features

• An on-demand platform allows for seamless testing (e.g., during probe trials, when the platform is usually removed), without interrupting the experiment to adjust/remove the platform.

• The focus here is to offer a step-by-step, economical alternative to otherwise costly, commercial on-demand platforms.

• Additionally, the protocol offers cost-effective ways of assembling the tank, preparing the testing environment, and implementing a testing protocol.

• We include testing strategies specifically developed for aged rats, as these animals are routinely used in our laboratories.

## Background

The Morris water maze (MWM) is widely considered a gold standard in behavioral neuroscience for assessing spatial learning and memory in rodents [1]. The basic protocol involves a circular pool filled with opaque water, where a submerged platform is hidden just below the surface. Rats or mice are placed in the water, where they must learn to locate the platform using spatial cues in the environment around the pool. With each learning (acquisition) trial, animals learn the location of the platform and will swim directly to it, demonstrating their spatial learning and memory abilities. The task is used extensively to assess the effects of various genetic, pharmacological, and environmental manipulations on cognitive function.

“Probe tests” provide converging evidence to that acquired by other behavioral measures. In the water maze, probe tests are classically implemented by removing the submerged platform from the tank; the experimenter measures the time the animal spends swimming in the area of the tank where the platform was previously located. Animals that recall the location of the platform spend more of their time near where the platform was previously located. A variant of the original MWM protocol assesses spatial memory over multiple probe trials, not just one. However, removing the platform during these probe trials results in these being extinction trials, since escape is not available. To address this, the probe trial can be modified so that the platform becomes available after a period of time. This procedure maintains response reinforcement in the probe trials as it is for the acquisition trials: go directly to the platform location and remain there. This modification to the protocol limits strategies like circling the wall of the tank until the platform is located, and instead promotes a spatial learning, hippocampus-dependent strategy [2]. Operationally, this requires the height of the platform to be adjustable (“on-demand”) such that it is just under the water surface during acquisition trials, or deep in the tank out of reach from the animal during probe trials [3].

The mechanisms controlling the height of on-demand platforms vary considerably, from cables and/or levers that are not visible to the rat to more technologically advanced methods that include remote-controlled devices. Notable advantages to the latter approach include greater consistency of raising/lowering timing, predictable speed, and minimizing disruption of behavior (e.g., experimenter visibility). However, the cost of such equipment is prohibitively expensive for modestly funded research programs [4]. Here, we developed a do-it-yourself remote-controlled on-demand platform, plus the tank, that costs less than a commercially available counterpart of the platform alone. The primary objective of this report is to disseminate a protocol for constructing a low-cost on-demand platform. However, in addition, we offer an abbreviated account of how we prepared the tank, set up the testing environment, ran a MWM protocol, tracked behavior, and analyzed data. As our aim was not to present a comprehensive MWM how-to, we direct the readers to other resources that, for example, provide more detailed protocols [5–8], detail the many variations of MWM protocols [6], describe its wide ranging applications [9], or offer parallel efforts to lower fiscal barriers to establishing MWM testing [4]. To validate functionality, we implemented our protocol to test whether our setup was sensitive to detect spatial learning and memory deficits among aged F344 rats.

## Materials and reagents

*Note: Information on vendors, model numbers, and links to purchase equipment are provided when possible. The list of materials used here can also be accessed in* ***Table S1***, *which also offers information about quantities of materials, prices, and abbreviated notes.*

**Platform**

1. Waterproof linear actuator (Progressive Automations, PA-10-4-450-N-12VDC, https://www.progressiveautomations.com/products/waterproof-linear-actuator-ip68m-for-outdoor-use?variant=31290949697603)

2. Mounting bracket (Progressive Automations, BRK-09, https://www.progressiveautomations.com/products/brk-09)

3. Control box and wireless remote (Progressive Automations, PA-33, https://www.progressiveautomations.com/products/pa-33)

4. Power supply (Progressive Automations, PS-20-12-67, https://www.progressiveautomations.com/products/ps-20-12-67)

5. Polycarbonate plastic sheet (buyplastic.com, PLA-033-029, https://buyplastic.com/products/abrasion-resistant-polycarbonate-ar2-plastic-sheet.html)

6. Flat white spray paint (Home Depot, model: 334021, https://www.homedepot.com/p/Rust-Oleum-Painter-s-Touch-2X-12-oz-Flat-White-General-Purpose-Spray-Paint-334021/307244842)

7. Stainless steel hex nut (Home Depot, model: 800051, https://www.homedepot.com/p/1-4-in-20-Stainless-Steel-Hex-Nut-4-Pack-800051/204274131)

8. Hex flat-head cap screws (Home Depot, model: 44048, https://www.homedepot.com/p/Hillman-1-4-in-x-1-1-2-in-Internal-Hex-Flat-Head-Cap-Screws-8-Pack-44048/204786023)

9. Stainless steel hex bolts (Home Depot, model: RTI2320604, https://www.homedepot.com/p/Robtec-1-4-in-x-2-in-Stainless-Steel-Hex-Bolts-5-Pack-RTI2320604/205710556)

10. Drawer liner (Amazon, model: DSL01-Charcoal-12x10, https://www.amazon.com/gp/product/B0BXSMJK86/ref=ppx_yo_dt_b_search_asin_title?ie=UTF8&th=1)

11. Flat head stainless steel sheet metal screw (Home Depot, model: 814871, https://www.homedepot.com/p/4-x-1-2-in-Phillips-Flat-Head-Stainless-Steel-Sheet-Metal-Screw-8-Pack-814871/204275449?irgwc=1&cm_mmc=afl-ir-2003851-1420157-EdgeBingFlow&clickid=TcOXWgwxLxyKRiEzHBxqD2W6UkCxFvRdRUHC1s0)

**Tank**

1. Round galvanized stock tank (Tractor Supply Co., SKU: 217713899, https://www.tractorsupply.com/tsc/product/countyline-round-galvanized-stock-tank-6-ft-w-x-2-ft-h-390-gal-capacity)

2. Hose bibb valve (Home Depot, model: 103-054EB, https://www.homedepot.com/p/Everbilt-3-4-MIP-or-1-2-FIP-x-3-4-in-MHT-Forged-Brass-Quarter-Turn-Hose-Bibb-Valve-103-054EB/205822403)

3. Metal filler (Amazon, UPC# 051115904516, https://www.amazon.com/Bondo-90451-Metal-ReinforcedFiller/dp/B010AGXBUO/ref=sr_1_1?dib=eyJ2IjoiMSJ9.35rS9mjvY4pgdC8G7bE1P6VbMBklyrtZka2juNV1F2Zw18XvnwYluhpdqIs1sHuaKOHZe4TxOTH40wQg35dohta2X98x81VGUR4OvwfZdPYhiTzbAAw4xJ7KVsnXpAX0_OVD4WjK8ebva8EfDE1ekJJK)

4. Kitchen and bath sealant (Home Depot, SKU# 1006465750, https://www.homedepot.com/p/DAP-AMP-Advanced-Modified-Polymer-9-oz-White-Kitchen-and-Bath-Sealant-00762/316997383?mtc=SHOPPING-BF-CDP-BNG-D24-024_002_CAULKS-NA-NA-NA-PLALIA-NA-NA-NA-NA-NBR-NA-NA-NA-2024&cm_mmc=SHOPPING-BF-CDP-BNG-D24-024_002_CAULKS-NA-NA-)

5. Rubber stopper, #0, 1-hole (Home Science Tools, SKU: CE-STOP0XA, https://www.homesciencetools.com/product/rubber-stopper-0-1-hole/?_ga=2.102155329.1984278044.1722538286-394657775.1722538286)

**Environment**

1. Curtain rod (Home Depot, model: AMB144FOHJ07, https://www.homedepot.com/p/Home-Decorators-Collection-72-in-144-in-Telescoping-1-in-Single-Curtain-Rod-in-Matte-Black-AMB144FOHJ07/303673688)

2. Pole sockets (Home Depot, model: 13609, https://www.homedepot.com/p/Everbilt-1-3-8-in-White-Plastic-Pole-Sockets-2-Pack-13609/203170044)

3. Curtain (Home Depot, model: DCV5096BE, https://www.homedepot.com/p/Pro-Space-50-x-96-Outdoor-Patio-Waterproof-Detachable-Sticky-Tab-Top-Porch-Decor-Privacy-Thermal-Insulated-Curtain-Beige-DCV5096BE/315746194)

4. Visual cues (endless options; see note in Procedure)

**MWM implementation**

1. Permanent marker (Amazon, model: 07888, https://www.amazon.com/Marks-Regular-Permanent-Markers-07888/dp/B000MIJ54M/ref=sr_1_6?dib=eyJ2IjoiMSJ9.K7-yaZSzK6sgBZLtmehNqwvX62jt20HbUxLT-zz-6jmTcD4XF9jGThes6VD6TGCgR8XNwAo92ncQChxJyAhVLnsrKLishBOboFiHiYtj0m6CHTrgwLwgM4aneV3qSa909d4OT5iBaKDjfHR9jy6Bi3Zf)

2. Camera (The Imaging Source, DFK 22AUC03, https://www.oemcameras.com/product/dfk-22auc03-htm/)

3. Lens (The Imaging Source, T2Z 1816 CS, https://dl-gui.theimagingsource.com/en_US/3a27d574-9c67-5e82-a776-6a94ac7bab6c/)

4. USB cable (Amazon, UPC #714133937095, https://www.amazon.com/gp/product/B07HH8KGG2/ref=ppx_yo_dt_b_search_asin_title?ie=UTF8&th=1)

5. Garden hose (Home Depot, Model #82-GHB-25-HD, https://www.homedepot.com/p/GrowGreen-3-4-in-x-25-ft-Expandable-Garden-Hose-82-GHB-25-HD/316267449)

6. Aquarium heater (Amazon, Model #jsmuwkwkP1218, https://www.amazon.com/Orlushy-Submersible-Thermostat-ThermometerFreshwater/dp/B08BPH67FW/ref=sr_1_5?crid=RT4D9N410BO0&dib=eyJ2IjoiMSJ9.riAWZAYvu6eE7LoKWI_p0paziiXTEFZJOxEac8x_LA_S6PUis6ryktXY4iTm2gJTUdFZoL3JekXPn5TVrJs79lyBZ7dsJ0LE_igvxD3DTKCspM-LkJVWZr)

7. Spring clamps (Amazon, Model #SP-04, https://www.amazon.com/EQUIPTZ-Spring-Clamps-inch-Non-Detachable/dp/B0BG8X1KGQ/ref=sr_1_2_sspa?crid=LBMQU6BZGRZG&dib=eyJ2IjoiMSJ9.lvYSPEvVFRsCipKIrvl7uhPyIzXiZSeHIYBxfAqmiP2IUHofGjE_TOYtnn6Flu6N3buMDi1bqxJkHE8LjNkQArKKpivqKXFuEWh9JtyC0xgntU0tTssBjWsAUApst)

8. Wireless presentation clicker (Amazon, model: 910-001350, https://www.amazon.com/Logitech-Professional-Presenter-PresentationWireless/dp/B002GHBUTU/ref=sr_1_3?crid=1BS74AFNU255V&dib=eyJ2IjoiMSJ9.d9kRR8HqvoKhQKJYbBjy4pIIDT8HCd37WJhpRNsb_qh98e0xbueBIgIgti45N6TInqaeb1pEtHiU4okIdSNJZFoc5Yq5cOlIxk54LY8RrRS-c-JKrKQnl)

9. Clamp mount (Amazon, model: RSX-157, https://www.amazon.com/Suptig-Gooseneck-Session-Action-Cameras/dp/B077QDKRC7/ref=sr_1_1?crid=E5FUSOFFWG8Z&dib=eyJ2IjoiMSJ9.TYIvBlMydtU_Q6_iNtWtJH_VeFHIEXvdVGJxQvjpBdHCuILVwVd7c5MmqtXDmMO6cG977M5oR0N4LBqX2LlkQsjNLRW7fD3kH5qLaFX1bwEJSWGSk5DoMm-KFINcx4SCP)

10. Tempera paint (Amazon, model: 214705, https://www.amazon.com/gp/product/B004DJ69MM/ref=ppx_yo_dt_b_search_asin_title?ie=UTF8&th=1)

11. Clamp lamp light (Amazon, model: #2-Pack Clamp Lamp Light, https://www.amazon.com/gp/product/B09J8H4MJ8/ref=ppx_yo_dt_b_search_asin_title?ie=UTF8&th=1)

12. Heat lamp bulbs (Amazon, model: Heat Lamp, https://www.amazon.com/Pyramid-250BR40-Reflector-Medium-Incandescent/dp/B07VWL8D33?th=1)

13. Aquarium fish nets (Amazon, model: QN3, https://www.amazon.com/Penn-Plax-Aquarium-Fish-Net/dp/B0002APXKA/ref=sr_1_1?crid=3OZZF0JT2BPWI&dib=eyJ2IjoiMSJ9.p6FBiO7aWslmlb8T5MWyuA.3s5Z9n2Gd0Ed3SmuEH39PHRx2vpVUFMD52OI1wX6eE&dib_tag=se&keywords=030172230042&qid=1720807319&sprefix=030172230042%2Caps%2)

## Equipment

**Platform assembly**

1. Drill

2. 1/2 in drill bit

3. 1/4 in drill bit

4. 1/16 in drill bit

5. Saw (to cut polycarbonate plastic sheet, e.g., circular saw, jigsaw, band saw)

6. Scissors

**Tank assembly**

1. Sandpaper

2. Drill

3. 3/4 in drill bit

## Software and datasets

1. Behavioral tracking software

*Note: While we were fortunate enough to obtain a temporary ANY-maze (Version 7.49) license through an independent loan program, purchasing a standard license of commonly used MWM tracking and analysis software packages (e.g., ANY-maze from Stoelting Co., Ethnovision from Noldus, Smart Video Tracking Software from Panlab, Med Associates, and HVS Image) can be prohibitively expensive for many labs. Fortunately, there is a growing number of open-source options available, including Wintrack [10], Pathfinder [11], MouBeAt [12], Rtrack [13], ezTrack [14], and LabCut [15], though we have not personally tested these potential options.*

## Procedure

**A. Platform assembly**

**Figure 1.**
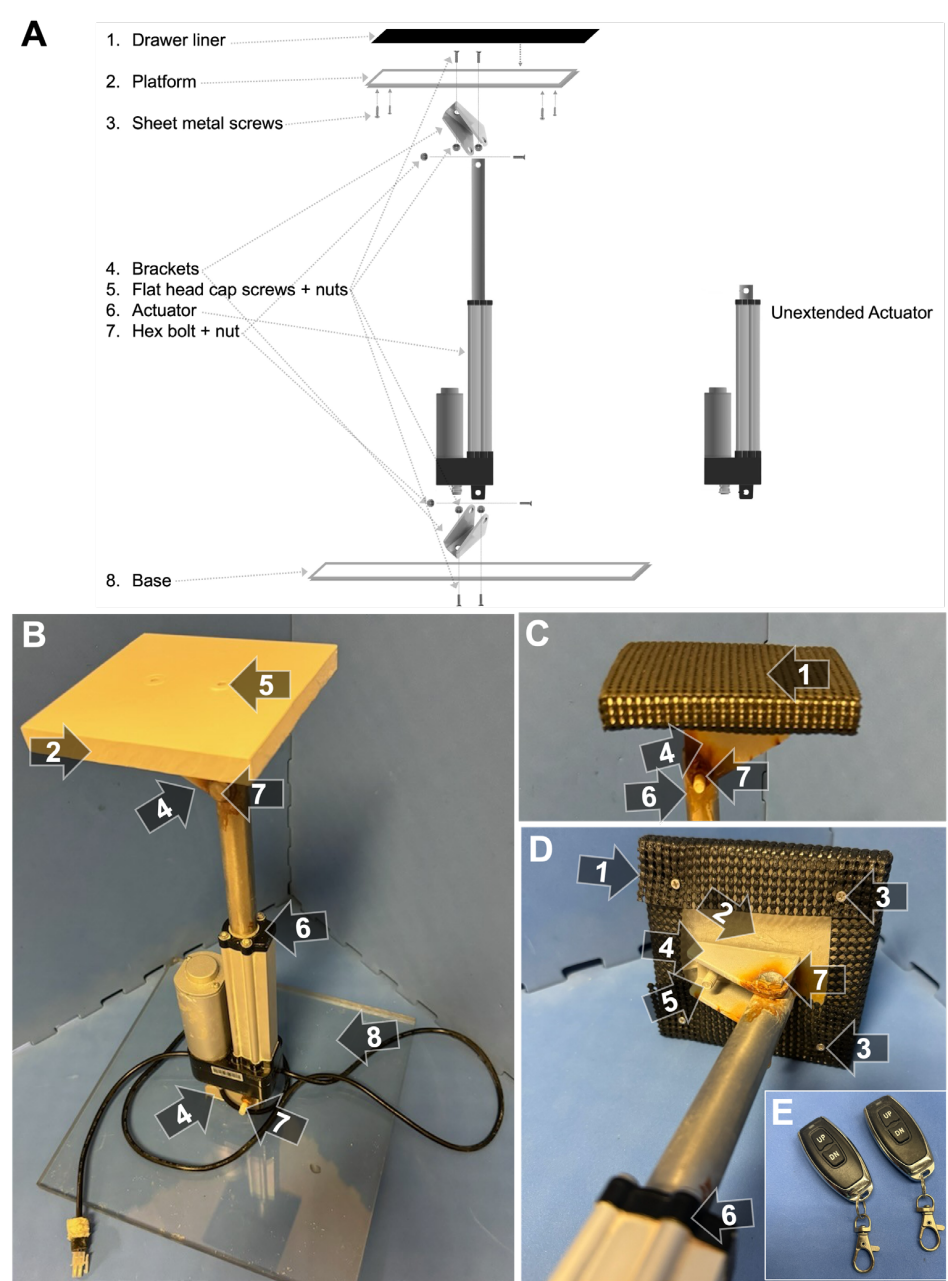
Platform assembly. (A) Schematic of platform assembly. (B) The on-demand platform, as used in acquisition and probe trials (i.e., in white). The polycarbonate plastic platform (2) was secured to a mounting bracket (4) by two flat-head cap screws (5). The mounting bracket was then attached to the actuator (6) by a hex bolt (7). The bottom of the actuator was attached to another bracket (4) with another hex bolt (7) and was fixed to a polycarbonate plastic base (8) via two more flat-head cap screws (5). (C) Top view of the platform as used in cue trials, where a piece of black drawer liner (1) was added for the purpose of enhancing visibility (i.e., increasing color contrast) and enhancing grip potential for rats while the platform is elevated. (D) Bottom view of the platform, as used in cue trials. Four sheet metal screws (3) were secured to each corner of the platform underside to hook the drawer liner to. (E) Wireless remotes that raise and lower the platform.

The critical piece of equipment for our on-demand platform was a waterproof linear actuator (Figure 1A–C, item #6) with a stroke length of 10.16 cm (4 in.). In addition, we purchased wireless remotes (Figure 1D), a power supply, and two mounting brackets (Figure 1A–C, item #4) from the same manufacturer. See Video 1, which shows the completed platform being raised.

**Video 1.**
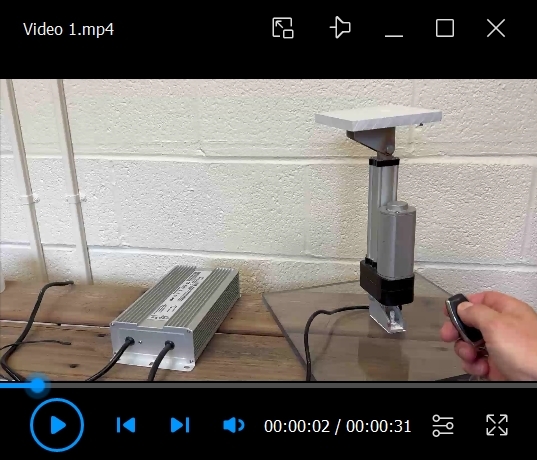
Raising and lowering of the actuator arm. Note that the remote-controlled raising and lowering of the actuator generates minimal sound.

1. Use a saw to cut a 12 cm × 12 cm piece of polycarbonate plastic for the platform (Figure 1A, B, D; item #2).

2. Use a saw to cut a 30 cm × 30 cm piece of polycarbonate plastic for the base (Figure 1A, B, item #8).

3. Center the mounting bracket (Figure 1A–D, item #4) on the *platform* and drill two 1/4 in holes through the plastic that align with the bracket holes. On top of the platform, drill two shallow 1/2 in holes on top of those holes to house the screw heads. Attach the bracket using the hex flat-head cap screws (Figure 1A, B, D; item #5) and stainless steel hex nuts.

4. As done in the previous step, center the mounting bracket on the *base* and drill two 1/4 in holes through the plastic that align with the bracket holes. On the bottom of the base, drill two shallow 1/2 in holes on top of those holes to house the screw heads. Attach the bracket using the hex flat-head cap screws and stainless steel hex nuts.

5. Spray paint all surfaces of the platform and screw caps white.

**Caution:** Use in a well-ventilated space to avoid inhaling harmful fumes.

6. On the underside of the platform, drill four shallow pilot holes ~2 cm from each corner using a 1/16 in drill bit. Do not drill all the way through.

*Note: Visual cue trials, where the platform is above the water surface and thus visible to the rat, are typically implemented to ensure that MWM performance is based on spatial learning and memory capacity and not affected by visual and/or motor ability. During these trials, two pieces of black drawer liner were attached to the platform by wrapping them around each side and attaching them to four screws on the undersurface of the platform, creating a high-contrast (i.e., visible), grippable surface for the rats (Figure 1A, C, D, item #1). Since our laboratory routinely tests older rats, this design decision took this into account. More specifically, to accommodate for age-related decreases in muscle strength [16] that could affect their ability to mount the platform during cue trials, we specially chose a material with ample gripping. Other simple, low-cost material options include black trash bags or silicone mats, though these alternatives do not offer as much grip. The black color of the material is also of particular importance, as it maximizes color contrast and thus visibility against the white water during cue trials.*

7. Screw a sheet metal screw into each hole such that approximately half of the screw is sticking out (Figure 1A, D, item #3).

8. Attach the platform bracket and base bracket to the actuator (Figure 1A–D, item #6) using stainless steel hex bolts and stainless steel hex nuts (Figure 1A–D, item #7).

9. Using scissors, cut two pieces of drawer liner (Figure 1A, C, D; item #1) to ~12 cm × 20 cm, fold over the platform, and hook them onto the machine screws (Figure 1A, D, item #3).

**B. Tank assembly**

1. Use metal filler per the manufacturer’s instructions to seal seams in the galvanized circular stock tank (1.82 m × 0.61 m). Sand down excess filler to create a smooth, homogenous tank wall.

**Critical:** One prominent welded seam runs vertically on the side of the stock tank. To ensure that rats do not use this ridge as a proximal cue, which would undermine the goal of the task, it is essential to create a tank wall with a consistent and even finish.

**Caution:** Use in a well-ventilated space to avoid inhaling harmful fumes.

*Note: For our MWM design, we also considered using an open-top plastic tank over the steel stock tank, but all options that we identified were considerably more expensive. Caution should be taken with some plastic varieties, as sizeable ridges in the tank walls may provide animals with sufficient leverage to facilitate escape. Two upsides of plastic tanks are that they lack a vertical seam, which eliminates the labor of concealing the welding ridge of steel tanks, and they do not require periodic repainting as do steel tanks.*

2. For draining the tank, drill a hole (3/4 in.) near the bottom of the tank (~2.5 cm) and install the turn hose bibb valve.

*Notes:*

*1. The valve should be as low as possible so that a maximal volume of water is drained when opened. However, be sure to allow sufficient room to connect the hose.*

*2. You may want to choose a location that is close to a drain.*

3. At another location near the base of the tank, drill an additional hole (~25 mm) to run the electrical wires for the platform.

*Note: You may want to choose a location that is close to an electrical outlet.*

4. To make a watertight seal around the electrical wire, use a standard rubber stopper for large test tubes (17 mm top diameter; 13 mm bottom diameter) with one hole in the center. Lead the wire through the hole by making one slice down the side of the stopper with a razor blade all the way to the center hole, and then slip the wire into the hole. Insert the plug into the hole from the inside of the tank.

5. To prevent leakage, apply kitchen and bath silicone sealant around both the rubber stopper and on the ridge along the bottom of the tank as necessary.

6. Apply spray paint to the top ~30 cm of the interior of the tank (Figure 3A).

**Caution:** Use in a well-ventilated space to avoid inhaling harmful fumes.

7. Plug the cord sticking out of the tank into the power supply and plug the power supply into an electrical outlet.

**C. Environment**

See Video 2, which shows the room that is ready for testing.

**Video 2.**
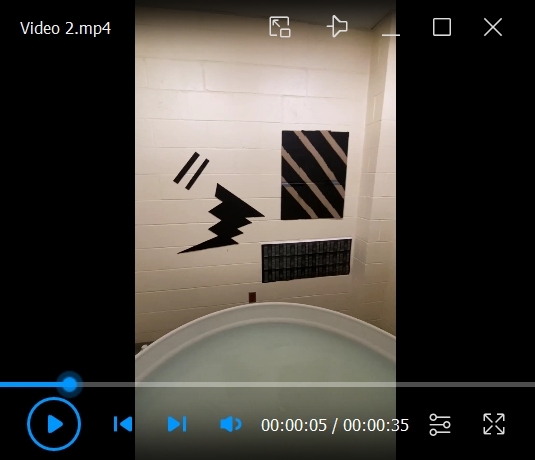
Platform, tank, and environment ready for testing. Note that the water is opaque and the platform is slightly submerged.

**Figure 2.**
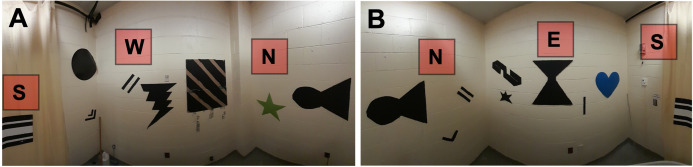
Testing environment. (A) Panorama of the western half of the room, containing various distal spatial cues. (B) Panorama of the eastern half of the room, containing various distal spatial cues.

1. Add visual cues, as necessary, to the walls surrounding the tank (Figure 2A, B).

*Note: Since our MWM was located in a small room (2.5 m × 2.5 m) lacking endogenous visual cues, we added several distal cues that were placed semi-randomly on each wall. Our cues were made of thick poster board cutouts (with a glossy surface that could be wiped down) of various shapes and sizes that were spray-painted black. For quick, easy, and quiet repeated access to the room during testing, we placed a large curtain of a similar color to the walls over the door. A telescoping curtain rod and pole sockets were used to hold it in place, and one additional black cardboard cue was hung from the curtain rod. We have observed that too few cues (one per wall) may promote a cue strategy that does not require intact hippocampal function (data not shown) rather than a spatial, hippocampus-dependent strategy, as also suggested by earlier studies [17]. If adding spatial cues is necessary to a testing environment, it is therefore advised to err on the side of “too many” [6]. Rooms available in other laboratories may have sufficient visual cues such that additional extramaze cues are unnecessary. Although we used black cardboard for our distal cues, the specific shapes and colors used were largely arbitrary, and any number of low-cost materials may also be used—from metal, wood, or poster paper to old conference posters or last year’s wall calendar.*

2. Install the camera.

*Note: Video of rodent behavior was acquired using a 2U Series monochrome industrial camera equipped with a T2Z 1816 CS vari-focal lens (The Imaging Source, Charlotte, NC). When discussing which camera to use for our MWM setup, we also considered several different low-cost webcams. As neither a color camera nor a high-resolution camera was necessary, we decided to use a low-resolution black and white camera, which offered smaller video files than several others that we considered. Cameras with a fisheye effect should be avoided, as they distort path length measurements. While we provide the specific mounting equipment that we used, it may be necessary to tailor this step to your specific needs. We attached the camera to a pipe in the ceiling using a clamp mount attached to a flexible gooseneck arm. A USB 2.0 type A cable connected the camera to the computer, which was placed just outside the room containing the MWM. Since we needed to run a long cord through the ceiling and into the room next door, we elected to use a camera that transmits data via USB1/2 cables, as they generally permit longer cable lengths.*

**D. MWM implementation**

We adopted a validated version of the MWM that has been previously described in detail [18] and is therefore not outlined here. Briefly, animals were handled for five consecutive days prior to testing, aimed at reducing the amount of stress during MWM testing. Since F344 rats are white, as was the water in the tank (described below), we ensured that tracking software could detect rats by using black non-toxic permanent markers to color each rat’s head during each day of handling. The testing phase of our protocol included sparse training, with three trials/day for eight consecutive days. The test duration maximum was 90 s per trial, and if the animal did not reach the platform by the end of the trial, it was guided to the platform, where it rested for 20 s. The animal was then removed and allowed to rest for an additional 40 s. Intermittent probe trials (last trial every other day) were implemented to track spatial bias development, where the platform was lowered to a depth out of reach by the rats via the remote control (Figure 1E). After 30 s, the platform was raised via the remote control (Figure 1E), and testing continued for 60 s, or until the rat reached the platform. As in acquisition trials, if the animal did not reach the platform by the end of the trial, it was guided to the platform, where it rested for 20 s. For all testing, to minimize the delay between the start of the trial and the start of the recording, we used a wireless presentation clicker to initiate testing immediately after the rat was placed in the tank. Finally, as discussed above (step A6, note), one day after the last day of MWM testing, rats underwent a single visual cue session consisting of six trials (30 s/trial), where the platform covered in black drawer liner was visible above the water surface. The following steps outline the steps required to prepare the tank and platform for testing.

1. Fill the tank to ~10 cm below the tank’s rim.

*Note: Ensure that the platform surface is 2 cm below the water surface on each day of testing.*

**Figure 3.**
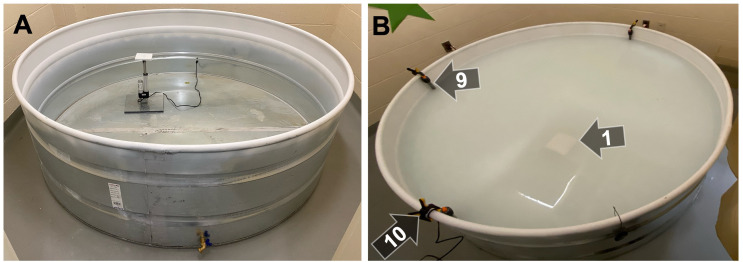
Tank prior to testing. (A) The tank prior to filling, showing the placement of the cord exiting the tank through the rubber stopper. (B) To maintain an optimal water temperature, three 500 W aquarium heaters (9) were clamped (10) to the edge of the tank. Clamps and heaters were removed immediately prior to testing each day and replaced immediately after testing each day. To prevent rats from seeing the submerged platform, white acrylic paint was added to the tank, resulting in an opaque water color.

2. To obscure visibility of the submerged platform, add enough white acrylic paint to create an opaque color (~1 L) (Figure 3B). Add more paint as needed prior to each day of testing.

*Note: Since we primarily use rats with white fur, we considered constructing a black tank and platform with black water [19]. Although we opted for a white platform, tank, and water, we presume that the black version would yield similar results. By darkening rats’ heads with a permanent marker during handling, the tracking software was capable of successfully tracking behavior. Alternatives to the permanent marker that we also considered were hair dye and veterinary markers. We find that white tempera paint works well to maintain the white opaque water color, though powdered milk is another common and cost-effective alternative.*

3. To maintain the temperature of the water in the tank at 24 °C ± 1, clamp three 500 W fish tank heaters (Figure 3, item #9) to the side of the tank using spring clamps (Figure 3, item #10). Heaters and clamps were removed during all testing.

*Note: Although this step is important for all MWM protocols, note that thermoregulation is particularly blunted in aged rats [20], and MWM performance can be negatively affected as a result [21]. In our experience, when these specific heaters were set to maximum, water temperature adjustments were rarely necessary prior to testing.*

4. Immediately following each day of testing, use heat lamps to maintain animal body temperature (~20 min) and ensure that animals are not shivering when returned to the animal colony.

5. Use a small fish net to remove feces from the tank after each trial.

## Data analysis

As the focus of this protocol is the construction of the platform, and this data analysis has previously been described elsewhere [18], details are not outlined here.

1. Calculate the *search error*.

*Note: This value reflects how efficiently each animal moves from the start position to the platform, where lower scores are better [18]. This measure implements a correction such that trial performance is relatively unbiased by differences in distance to the goal from the various start locations, as well as by swim speed.*

2. Calculate the spatial learning index (LI) score.

*Note: This was derived for each rat by using the mean search error sum across probe trials 2, 3, and 4 that was weighted such that earlier probes had a greater impact on score (weighted 1.26, 1.43, and 1.43, respectively) [18,22]. Lower LI scores reflect better performance. Although we also report other commonly used measures of MWM performance (i.e., acquisition search error, probe search error, and percentage of time in goal quadrant), the LI score is advantageous in that it 1) condenses complex behavioral data into a single value reflecting performance, 2) facilitates within-group comparisons and correlations with neurobiological markers or other behavioral measures, and 3) detects subtle differences between individuals [18,22].*

Statistical analyses were performed using GraphPad Prism, version 10 (San Diego, CA). Comparisons between age groups for acquisition search error, probe search error, and percentage of time in goal quadrant were conducted using repeated measures 2-way ANOVAs (age × training session or probe trial), and Sidak multiple comparisons tests were conducted where appropriate. Unpaired t-tests were used to compare LI scores between age groups, and the relationship between LI scores and cue trial path lengths was evaluated by using the Pearson correlation test. Given the small sample size of this pilot study, we did not test for sex differences, and data for males and females were combined.

## Validation of protocol

**Animals**

A total of 13 F344 rats obtained from the National Institute on Aging colony were tested in this pilot study, which included 2 young males (6 months), 2 young females (6 months), 5 aged males (23 months), and 4 aged females (23 months). Animals were ear-marked for identification, maintained on a 12-h ON, 12-h OFF light schedule, and fed Teklad rodent diet ad libitum (7012; Envigo, Indianapolis, IN). All experimental procedures were approved by the Loyola University Maryland Institutional Animal Care and Use Committee.

**Results**

**Figure 4.**
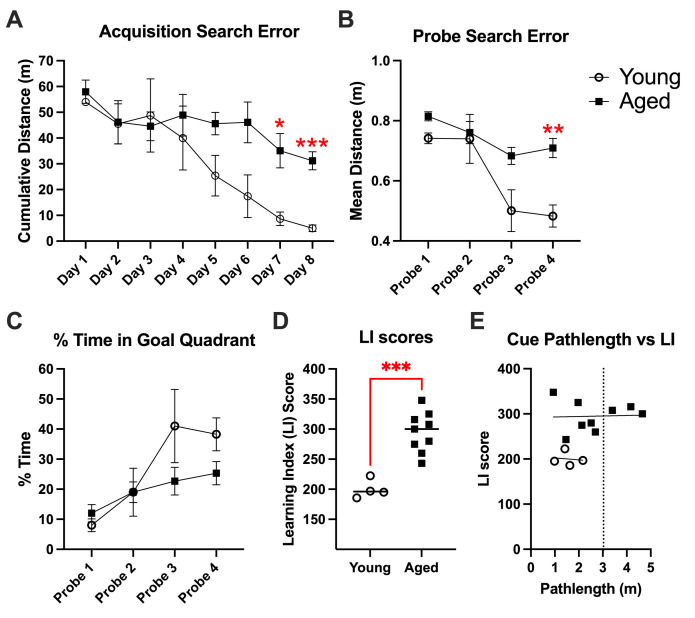
Morris water maze (MWM) data of young and aged F344 rats. (A) Performance during acquisition trials, as assessed by cumulative search error. Data points are averages across animals and trials on each day of training (mean ± SEM). (B) Performance during probe trials, as assessed by search error. Data points are averages across animals on each probe day (mean ± SEM). (C) Performance during probe trials, as assessed by the percentage of time spent in the goal quadrant. Data points are averages across animals on each probe day (mean ± SEM). (D) The average proximity for the four probe trials was combined into a single LI score. (E) Cue path length of young and aged animals plotted against LI scores. The vertical dashed line represents three standard deviations above young performance, which was used as the exclusion cutoff for animals that may have had sensorimotor or motivational deficits.

A repeated measures ANOVA (age × training session) performed on cumulative search error during acquisition trials (Figure 4A) showed that rats improved over the course of training [F(4.34, 47.73) = 6.547, p = 0.0002] and that there was a main effect of age on training trial performance [F(1,11) = 6.06, p = 0.03]. Post-hoc comparisons revealed that aged rats performed significantly worse than young rats on day 7 (p = 0.03) and day 8 (p = 0.003).

Probe trials, where the platform was lowered to a depth that rats could not escape for a duration of 30 s, were interpolated throughout the training procedure and assessed spatial bias for the quadrant where the platform was previously located. As in acquisition trials, a repeated measures 2-way ANOVA of search error during probe trials (Figure 4B; age × probe trial) confirmed that all rats improved over the course of training [F(2.2,23.8) = 15.2, p < 0.0001] and that there was a main effect of age [F(1,11) = 11.45, p = 0.006]. We also observed a significant interaction for search error, where aged rats improved to a lesser degree than young rats over the course of probe trials [F(3,33) = 3.5, p = 0.03]. Post-hoc comparisons indicated that aged rats performed significantly worse than young ones by Probe 4 (p = 0.0075). As a secondary way to assess spatial bias during probe trials, we compared the percentage of time rats spent in the correct quadrant (Figure 4C). Those analyses yielded similar results, showing that all rats improved over the course of training [F(2.23,24.85) = 9.95, p < 0.0005]. This measure did not reach significance for an age effect, though we observed a trend in the same direction (p = 0.19).

Next, we compared LI scores (Figure 4D), which are computed from the ongoing distance (measured 10×/s and a mean was taken every second) between the platform location and the animal. An unpaired t-test indicated that aged rats were significantly impaired compared to young rats [t(11) = 5.4; p = 0.0002], where all LI scores of aged rats were higher than all young rats. These findings differ from an earlier MWM assessment in F344 rats, showing that approximately half of aged rats performed on par with young rats [23]. The small sample size in this pilot experiment may contribute to this discrepancy.

To assess whether sensory (visual ability), motor (swimming ability), or motivation deficits might contribute to MWM performance indices, “cue” trials were conducted, where the platform was clearly visible (Figure 4E). These trials require sensory, motor, and motivation but not intact spatial memory. We found that path length was not correlated with MWM performance among either aged animals (r = 0.04, p = 0.92) or young animals (r = -0.14, p = 0.86). Three aged animals performed more than three standard deviations beyond young performance, which we established as our exclusion criteria.

**Discussion**

A number of earlier studies that similarly compared MWM performance of young and aged F344 rats provided a means by which we could assess the validity of our newly established MWM. Overall, as in many other studies, young and aged animals’ proximity to the platform on the first day of testing was comparable, and both groups of animals showed improvement over the course of training and probe trials, though aged animals exhibited a slower rate of acquisition than young animals [23–25]. However, comparisons of LI scores revealed that all aged animals were impaired compared to their young counterparts. These data are consistent with at least one study in F344 rats, where the data similarly showed limited interindividual variability in cognitive outcome in aging [25]. However, studies utilizing LI scores to compare MWM performance, which was optimized to detect subtle age-related spatial memory deficits [18], showed substantial interindividual variability in cognitive outcome in aging. More specifically, one study found that ~53% of aged rats performed within the range of young animals [23], and another study reported that ~39% performed on par with young rats [24]. While the comparatively smaller cohort tested here may account for some of this discrepancy, one other possibility is that the animals tested here were 24 months of age, compared to 22 months.

## General notes and troubleshooting

**General notes**

1. While the specific materials and methods described here were optimized for our laboratory space, budget, and specific research interest in cognitive aging, we considered several alternative strategies that might be more suitable for other laboratories, which are discussed in the relevant protocol sections above. It is also noteworthy that researchers working with mice can adopt the same or similar materials. Modifications should account for differences in size, physiology, behavior, and cognitive function between the two species. The most notable modifications include the reduction of tank and platform diameters by roughly half (tank to ~90–120 cm; platform to 15–20 cm). As mice are more vulnerable to hypothermia than rats, water temperature must be carefully controlled, among other considerations (e.g., heating pads, prewarmed holding cages, drying off mice after testing) [15]. The maximum trial duration is also often shorter for mice (60 s vs. 90–120 s), and inter-trial intervals are sometimes longer in mice [6]. Given the substantial variability in water maze performance among mouse strains [26], a comprehensive review of existing literature on the chosen strain is strongly recommended.

2. Minimal maintenance is required for the equipment. At the conclusion of testing, the tank should be fully drained, the platform thoroughly rinsed, and the walls and bottom of the tank hosed down. Due to the use of seemingly durable, high-quality components, routine replacement is not expected, aside from occasional changes to the drawer liner covering the platform.

3. The total cost of ~1,800 USD for our MWM design includes effectively everything—from core components like the tank, platform, and camera—to items more easily overlooked like heat lamp bulbs and a small fish net to scoop out feces from the tank (see the Supplies listed above and **Table S1**), with the exception of a specific tracking software (see notes in Software section).

4. The MWM is a cornerstone of behavioral neuroscience, thanks in part to its reliability and ability to facilitate the identification of the neural basis of spatial learning and memory. Despite its prominence as a tool for researchers studying cognition, brain function, and related disorders, the cost of commercially available MWM equipment is often prohibitively expensive to implement. We hope that disseminating low-cost strategies to construct MWM equipment in-house, such as that described here, will help to accelerate biomedical discovery. Our focus on cognitive aging serves as an illustrative example, and these data provide us with a foundation for discovering mechanisms underlying cognitive outcomes in aging. The MWM has been used in an impressive variety of contexts, and the specific strategy implemented here can similarly be tailored to assay spatial learning and memory in laboratory rodents in a wide range of applications.

## Supplementary information

The following supporting information can be downloaded here:

1. Table S1. Supplies: Company, item number, description, quantity, price, corresponding number in Figure 1, and notes
